# How do junior medical officers use online information resources? A survey

**DOI:** 10.1186/s12909-016-0645-x

**Published:** 2016-04-22

**Authors:** Heng Teck Chong, Michael James Weightman, Peranada Sirichai, Alison Jones

**Affiliations:** Department of Paediatrics, University of Adelaide, 72 King William Road, North Adelaide, South Australia 5006 Australia; Royal Adelaide Hospital, North Terrace, Adelaide, South Australia 5000 Australia; Department of Medicine, University of Adelaide, Adelaide, South Australia 5000 Australia; Westmead Hospital, Cnr Hawkesbury Road and Darcy Road, Sydney, NSW 2145 Australia; South Australian Medical Education and Training Unit, Department of Health, 11 Hindmarsh Square, Adelaide, South Australia 5000 Australia

**Keywords:** Information resources, Junior doctors, Medical education

## Abstract

**Background:**

Online information resources function dually as important learning tools and sources of the latest evidence-based recommendations for junior medical officers (JMOs). However, little is currently known about how JMOs utilise this information when providing care for their patients. This study aimed to examine the usage and experience of online information resources amongst JMOs in South Australia to ascertain (i) the type of resources accessed, (ii) the frequency, (iii) the intended purpose, and (iv) the perceived reliability.

**Methods:**

A survey instrument using multiple choices, five-point Likert scales and free-text comments was developed and distributed through SurveyMonkey to South Australian JMOs between 1 May 2014 and 30 June 2014.

**Results:**

Of the 142 surveyed, 100 JMOs (70.4 %) used online information resources as their first approach over all other resources available. JMOs overwhelmingly (94.4 %, *n* = 134) used online information resources at least once per day, with the most frequent purpose for use being information regarding prescription medication (82.4 %, *n* = 117, reported ‘very frequent’ use). JMOs stated online resources were necessary to perform their work and, of the different types of information accessed, they rated peer-reviewed resources as the most reliable.

**Conclusions:**

JMOs strongly rely upon online clinical information in their everyday practice. Importantly, provision of these resources assists JMOs in their education and clinical performance.

**Electronic supplementary material:**

The online version of this article (doi:10.1186/s12909-016-0645-x) contains supplementary material, which is available to authorized users.

## Background

The Internet has caused a significant shift in the way doctors source clinical information, with an increasingly greater amount of medical knowledge now available and being accessed online [1]. The importance placed on evidence-based practice ensures that the Internet has become an essential tool as it contains the most up-to-date information in vast quantities [2]. With the field of medicine being a continually renewing and expanding body of knowledge and clinical practice, it is challenging for all doctors to keep abreast of the latest evidence and therapeutic recommendations. This challenge is particularly so for junior medical officers (JMOs), who do not have the experience and knowledge-base of their senior colleagues [3].

This increased role for information technology appears to be particularly embraced by junior doctors [4], who are typically younger and more intuitive with technology. Given their limited clinical experience, it is reasonable to expect that online clinical information resources would serve to equip JMOs with additional knowledge to assist them in diagnosing and managing patients’ conditions. Such sources would offer an important mechanism for ongoing education, as well as assisting in the clinical and diagnostic process. From clarifying a medication dosage to looking up clinical approaches for complex medical presentations, evidence-based platforms such as *electronic Therapeutic Guidelines* [5] or *UpToDate* [6] are designed to help doctors to make the best decisions for their patients.

Many hospitals provide open access to various clinical resources online through hospital servers for their doctors to use in their work. This represents a significant financial commitment to assist hospital doctors to achieve best practice. However, little is currently known about the way junior medical officers rely on such clinical information resources or how they use these resources when providing health care. This is particularly important as much misinformation exists online from non-peer reviewed sites, and this could have adverse patient outcomes if inappropriately relied upon [7, 8].

The aim of this study was to evaluate how South Australian hospital JMOs used the clinical resources online and their experience of them. It aimed to ascertain (i) the type of online information resources accessed, (ii) the frequency of access, (iii) the intended purpose for consulting the resource, and (iv) the perceived reliability of the information retrieved. This information may have implications in guiding how financial resources are allocated for JMOs, improvement of training experience for JMOs and, potentially, maximising patient outcomes.

## Methods

### Survey

A 16-question online survey instrument was developed by the authors to address the stated aims of the investigation. This included a combination of multiple choice questions, five-point Likert scales and free-text comment boxes. The survey questions were arranged into five distinct sections: demographics, types of resources used, resource reliability, frequency/ease of access, and general comments. These sections contributed to addressing the four key aims of the study, which were (i) the type of resources accessed, (ii) the frequency of use, (iii) the intended purpose, and (iv) the perceived reliability. The questions were uploaded onto the SurveyMonkey website (SurveyMonkey, Palo Alto, CA, USA) for distribution and data collection. The full survey used in this study is included in the Additional file 1.

### Participants

Junior doctors were recruited for voluntary participation in this study, which solely consisted of completion of the survey as described above. The inclusion criterion for each participant was a requirement to hold current employment as an Intern or prevocational Resident Medical Officer (RMO) in a South Australian public hospital or affiliated training location. In 2014, the Department of Health in South Australia employed 278 Interns and 271 RMOs, totalling 549 Junior Medical Officers across the different Local Health Networks [9].

Participants were recruited via hospital email contact lists, the South Australian Junior Medical Officers Forum newsletter and verbally at face-to-face hospital training sessions. Potential participants were provided with a URL which would direct them to information about the study with a link for commencement of the survey. The survey was open for the period between 1 May 2014 and 30 June 2014 and took approximately seven minutes to complete.

Participation in this study was strictly voluntary. Detailed explanation was provided prior to the commencement of the survey regarding the purpose of the study and the way the collected information would be used. Informed consent was implied by the potential participant clicking the option to proceed to answering the questions after reading the information slide. Participants had the option to withdraw consent at any time prior to submitting responses.

All information was anonymous, although participants were given the option to submit their contact details to enter a prize draw for a tablet device. If a participant provided this information, the data were de-identified prior to analysis.

### Analysis

All data were computed using Microsoft Excel 2010 (Microsoft Corporation, Redmond, WA, USA). Results are presented either as percentage or as mean ± standard deviation.

## Results

In total, 142 junior doctors (74 Interns and 68 RMOs) were surveyed from multiple different sites across South Australian health services. Given that the total number of prevocational junior doctors in South Australia was 549, the response rate was 25.9 %.

### Types of resources accessed by junior doctors

When junior doctors were asked what their first approach would be to answer a clinical query, online information resources were the most frequent option selected. Indeed, 70.4 % (*n* = 100) of junior doctors replied that this would be their first approach a “few times each day” (Table 1). The next most preferred options were asking a senior colleague or a peer colleague, which were only pursued a “few times a day” by 50.7 % (*n* = 72) and 29.5 % (*n* = 42) of junior doctors respectively. Print textbook and original articles are the least likely first approach, with almost half of junior doctors using them less than once per week.

**Table 1:** “How often would each resource be your first approach when seeking clinical information?”

	Few times daily	Daily	Few times a week	Once a week	Less than once a week
Online information resources	100 (70.4 %)	27 (19.0 %)	14 (9.9 %)	1 (0.7 %)	0 (0.0 %)
Ask a senior colleague	72 (50.7 %)	43 (30.3 %)	23 (16.2 %)	3 (2.1 %)	1 (0.7 %)
Ask a peer colleague	42 (29.5 %)	38 (26.8 %)	35 (24.6 %)	13 (9.2 %)	14 (9.9 %)
Print textbook or original journal	4 (2.8 %)	9 (6.3 %)	25 (17.6 %)	36 (25.4 %)	68 (47.9 %)

Junior doctors were given a list of the online information resources available either through the South Australian Health online portal or were anecdotally reported to be used frequently by junior doctors. They were asked which in the list they had used in the month prior to the survey and almost all respondents had used *Australian Medicines Handbook* [10] (95.1 %, *n* = 135), *electronic Therapeutic Guidelines* [5] (94.4 %, *n* = 134) and *UpToDate* [6] (88.0 %, *n* = 125) (Table 2). Respondents were additionally given a free-text box to list other information resources they have accessed which were not present in the list shown in Table 3. The resources mentioned were *South Australian Perinatal Practice Guidelines* [11] (*n* = 3) and *DermNET* [11] (*n* = 2).

**Table 2:** List of common online information resources used by junior doctors in South Australia. The numbers and percentage of junior doctors who had accessed each resource in the month prior

Online Information Resources	Number	Percent
Wheeless’ Textbook of Orthopaedics [20]	7	4.9 %
Australian Injectable Drugs Handbook [21]	9	6.3 %
TOXINZ [22]	14	9.9 %
Subscription Publishing Databases	16	11.3 %
Best Practice [23]	25	17.6 %
Royal Children’s Hospital Guidelines [24]	33	23.2 %
PubMed or Google Scholar [25, 26]	49	34.5 %
eMedicine.com (Medscape) [27]	65	45.8 %
Wikipedia [12]	79	55.6 %
Mims Online [28]	85	59.9 %
Random Google search [13]	108	76.1 %
Local hospital clinical guidelines	109	76.8 %
Pharmaceutical Benefits Scheme [29]	113	79.6 %
UpToDate [6]	125	88.0 %
Australian Medicines Handbook [10]	134	94.4 %
Therapeutic Guidelines (electronic) [5]	135	95.1 %

**Table 3:** How often do junior doctors use online information resources?

Frequency	Number	Percent
More than once a day	117	82.4
Once a day	17	12.0
Once every few days	8	5.6
Once a week	0	0
Less than once a week	0	0
Less than once every few weeks	0	0

### Frequency of use

In addition to their preferred approach for addressing a clinical query, the respondents were also asked about how frequently they used such resources. Regarding online information resources, 117 of the 142 junior doctors surveyed (82.4 %) used these resources a few times daily, 17 (12 %) use them daily and eight (5.6 %) use them once every few days (Table 3). No response was received for “once a week”, “less than once a week” and “less than once every few weeks”.

Of the common online information resources specifically referenced in the survey, the frequency of usage was examined. Australian Medicines Handbook [10] was “used daily” by 59.9 % (*n* = 85) of all junior doctors (Table 4). Additionally, 40.1 % (*n* = 57) of junior doctors use electronic Therapeutic Guidelines [5] daily. Although local hospital clinical guidelines were used by 76.8 % (*n* = 109) of junior doctors in a month prior (Table 2), they were “often used” by 49.3 % (*n* = 70) and “used daily” by 28.9 % (*n* = 41) of junior doctors (Table 4). In contrast, while 88.0 % of junior doctors had used UpToDate [6] (*n* = 125) in the prior month, this resource was only used “daily” by 22.5 % (*n* = 32) of junior doctors. Research databases were only used “rarely” and “sometimes” by most junior doctors (26.8 %, *n* = 38, and 45.1 %, *n* = 64, respectively).

**Table 4:** Usage frequency of common online information resources

	Used daily	Often used	Sometimes used	Rarely used	Never used
Australian Medicines Handbook [10]	85 (59.9 %)	40 (28.2 %)	12 (8.4 %)	4 (2.8 %)	1 (0.7 %)
Therapeutic Guidelines (electronic) [5]	57 (40.1 %)	64 (45.1 %)	17 (12.0 %)	3 (2.1 %)	1 (0.7 %)
Local hospital clinical guidelines	41 (28.9 %)	70 (49.3 %)	26 (18.3 %)	4 (2.8 %)	1 (0.7 %)
Wikipedia or Google search [12, 13]	34 (23.9 %)	48 (33.8 %)	44 (31.0 %)	14 (9.9 %)	2 (1.4 %)
UpToDate [6]	32 (22.5 %)	62 (43.7 %)	33 (23.2 %)	11 (7.8 %)	4 (2.8 %)
Mims Online [28]	24 (16.9 %)	30 (21.1 %)	38 (26.8 %)	28 (19.7 %)	22 (15.5 %)
Research Databases	2 (1.4 %)	25 (17.6 %)	64 (45.1 %)	38 (26.8 %)	13 (9.1 %)

### Intended purpose

The purpose junior doctors had for accessing online resources was assessed via the respondents being presented with a list of common reasons for use and they were then asked how frequently they accessed the Internet for each reason. This employed a five-point Likert scale that ranged from “very frequently” to “never”. The survey showed that 82.4 % (*n* = 117) of junior doctors reported that, on average, they access online information resources for medication information “very frequently”. Therapy information was the next most common, where 54.2 % (*n* = 77) of junior doctors reported “frequently” seeking related information. Less common purposes were for choosing investigations, researching complex clinical presentations and clarifying diagnoses, where 56.3 % (*n* = 80), 52.1 % (*n* = 74) and 43.7 % (*n* = 62) of junior doctors respectively reported that they only “sometimes” seek this information for these purposes (Table 5).

**Table 5:** How frequently do JMO use online resources to seek information for each purpose?

	Very frequently	Frequently	Sometimes	Rarely	Never
Medication	117 (82.4 %)	25 (17.6 %)	0 (0.0 %)	0 (0.0 %)	0 (0.0 %)
Therapy	19 (13.4 %)	77 (54.2 %)	41 (28.9 %)	5 (3.5 %)	0 (0.0 %)
Investigations	4 (2.8 %)	36 (25.4 %)	80 (56.3 %)	22 (15.5 %)	0 (0.0 %)
Diagnosis	2 (1.4 %)	41 (28.9 %)	74 (52.1 %)	25 (17.6 %)	0 (0.0 %)
Complex clinical presentation	5 (3.5 %)	47 (33.1 %)	62 (43.7 %)	25 (17.6 %)	3 (2.1 %)

Participants were also invited to provide free-text responses at the conclusion of the survey. The majority of responses in this section stated that online information resources were considered necessary, and sometimes critical, to the job by the surveyed cohort of junior doctors.

### Perceived reliability

Using a scale from 1 to 5 where 5 is “most reliable” and 1 is “not reliable”, junior doctors rate electronic Therapeutic Guidelines [5] at 4.83 ± 0.03 and Up-to-Date at 4.65 ± 0.04. 118 of 142 junior doctors (83.1 %) rated electronic Therapeutic Guidelines as very reliable, while 94 (66.2 %) gave UpToDate [6] the same rating. Other online information resources assessed for reliability included print textbooks and journals (3.85 ± 0.07), Wikipedia [12] (2.47 ± 0.08) and random Google [13] search results (2.48 ± 0.08). All of these options were rated as being markedly less reliable.

## Discussion

Online information has become a significant source of knowledge to complement junior doctors’ ongoing education and learning. Previous literature has highlighted that online clinical resources are important for junior doctors as learning tools [14]. We found that the reliance on online clinical resources is overwhelming, as more than 80 % of junior doctors surveyed in the present study accessed them multiple times per day. When in a moment of doubt, the convenience of online information resources enables clinicians to readily seek and clarify the information they need. We expect the reliance on information resources to be more pronounced in junior doctors who are still in the steeper phase of learning at this early stage of their career.

Results from this study allude to the importance of reliable and valid online clinical resources for junior doctors to perform on the job. More than half of the respondents are first year doctors who, as part of their medical clerkship, gain the majority of their learning and experience through direct clinical contact with patients. In addition to promoting work-based learning, this has significant direct flow-on benefits to standards of healthcare. A previous investigation by Westbrook et al. [15] demonstrated both objectively and subjectively that the primary intended purpose of clinicians’ use of online information is for patient care, rather than self-directed study or research, and this use is particularly intense when a new patient is initially admitted.

Selected online information resources provide current information in contrast to textbooks. Indeed this study observed that print textbooks are the least preferred first option for junior doctors when seeking information. Previous studies have noted that most interns and residents do not frequently use research databases and original articles compared to online information resources and print textbooks [14, 16]. This trend was observed in our study as most junior doctors do not prioritise research databases and original articles as a first approach when seeking information. Our study shows that junior doctors show a significant inclination to access electronic resources, which is in contrast to an earlier study that found that many senior doctors (specialist consultants and general practitioners) reported that they did not have the required computing skills to efficiently search online and therefore used it less commonly than printed texts [1]. This earlier study also found that senior doctors had a distinct preference for sourcing information by asking a colleague or using a printed text [1]. The findings of the present survey indicate that junior doctors are leading the trend towards using technology in answering clinical questions.

Our results show that Australian Medicines Handbook [10], Therapeutic Guidelines [5], UpToDate [6] and local hospital clinical guidelines are the most commonly used and most reliable sources available to junior doctors. This suggests that this group of clinicians generally access online information resources in order to answer a specific clinical question. Particularly in this survey, medication and therapy information are the most common information junior doctors seek, which is consistent with previous studies [17, 18].

The use of high-quality evidence from online literature has also been shown to be beneficial for solving clinical quandaries. Westbrook et al. [19] conducted an experiment where experienced clinicians were asked to provided answers to questions relating to various clinical scenarios before and after the use of online information. The use of the online resources was found to significantly improve the accuracy of the answers provided, indicating the potential benefit to patients if their doctors are able to consult such sources when looking after them. Indeed, when surveyed, clinicians reported that they had personally experienced online information resources improving patient care [15]. The benefits could be expected to be even more pronounced for junior doctors who, unlike the senior clinicians in this study, do not yet have the same wealth of experience of medical practice to assist them. Given the uptake of use amongst junior doctors as shown in our study, hospitals should provide the infrastructure for junior doctors to access online information resources.

There are some limitations to our study. The actual usage records of junior doctors were not directly examined. Instead the data analysed were junior doctors’ subjective self-reports and this would be limited both by their recall, but also by a potential bias to the needs of the current clinical rotation they were undertaking. The survey instrument designed was specifically brief and intended to maximise responses in a cohort that is notoriously time-poor. This was successful, as a quarter of potential respondents agreed to provide the information anonymously. Care should also be taken to generalise the results to other health jurisdictions outside South Australia, as the online clinical resources available could be different in terms of hospital subscriptions and ease of access.

Green et al. [17] has previously shown that a junior doctor develops two clinical questions for every three patients encountered including diagnosis and therapy [17]. Alarmingly, only 29 % of the questions were ever pursued for an answer. That study was conducted in a time where print medical textbooks were the main media for information and learning. The results shown in the present survey will be heartening for clinical teachers as junior doctors today are actively seeking answers for questions they have relating to patient care. Online clinical resources can effectively integrate education with everyday clinical practice. Additionally, a better understanding of the purpose junior doctors have for using the resources and the specific nature of the resources accessed could be beneficial to clinical teachers aiming to address potential education gaps.

## Conclusion

This study indicates for the first time that online information resources are strongly relied upon by junior medical officers in their everyday clinical practice. This is part of a generational shift from print media to Internet-based information. Online clinical resources are important learning tools and sources of the latest evidence-based recommendations for JMOs. These are highly valued by the doctors who use them to make informed decisions for their patients. We recommend the provision of these resources as essential for JMOs’ clinical performance and patient safety.

### Ethics approval and consent to participate

Ethical Approval for this study was obtained from the SA Health Human Research Ethics Committee (ref: HREC/15/SAH/24). All participants read a ‘participant information’ before taking part and completion of the survey was taken as implied consent.

### Availability of data and materials

Survey questionnaire is listed in the Additional file 1. The complete dataset can be requested from the first author.

## Additional file

Additional file 1: JMOs and Clinical Information Resource Survey. (PDF 235 kb)

## Abbreviations

JMO: junior medical officer
