# Supplementary material for: How do junior medical officers use online information resources? A survey
Source: BMC Med Educ. 2016 Apr 22;16:120. doi: 10.1186/s12909-016-0645-x (PMC4840860; doi:10.1186/s12909-016-0645-x)
Supplement: Additional file 1: — JMOs and Clinical Information Resource Survey. (PDF 235 kb) [file 12909_2016_645_MOESM1_ESM.pdf]

JMOs and Clinical Information Resource Survey

Junior hospital doctors have access to a wide range of clinical resources in the context of an ever expanding body of evidence and therapeutic recommendations. Junior doctors often rely on online information to make clinical decisions.

The purpose of this survey is to better understand how junior doctors use online clinical resources, with the results to help shape future decisions about availability of resources. This survey closes on 30 June 2014.

Please tell us a little bit about yourself

1. Which category of junior doctor best describes your current practice?

- ☐ Intern
- ☐ Resident (PGY2+)
- ☐ PGY2+ (enrolled with specialty college)

2. Which age bracket do you fall into?

- ☐ 20-29
- ☐ 30-39
- ☐ 40-49
- ☐ 50+

3. What type of medical degree did you attain?

- ☐ Undergraduate (Australian or New Zealand trained)
- ☐ Graduate-Entry (Australian or New Zealand trained)
- ☐ Overseas Qualifications

Online Clinical Resources Usage

4. How often would each of the following resources be your first approach when seeking clinical information? (Please tick)

	Few times daily	Daily	Few times a week	Once a week	Less than once a week
Ask a peer colleague	☐	☐	☐	☐	☐
Ask a senior colleague	☐	☐	☐	☐	☐
Print textbook or journal	☐	☐	☐	☐	☐
Online clinical resources	☐	☐	☐	☐	☐

5. How often do you use online clinical resources?

- ☐ Once a day
- ☐ Once every few days
- ☐ Once a week
- ☐ Less than once a week
- ☐ Less than once every few weeks

6. How frequently do you use online resources to seek information regarding the following categories of clinical decisions?

	Very frequently	Frequently	Sometimes	Rarely	Never
Investigations	☐	☐	☐	☐	☐
Diagnosis	☐	☐	☐	☐	☐
Therapy	☐	☐	☐	☐	☐
Medication	☐	☐	☐	☐	☐
Complex clinical presentation	☐	☐	☐	☐	☐

7. From the following list of resources, tick those that you have used in the LAST MONTH:

- ☐ Mims Online
- ☐ Australian Medicines Handbook
- ☐ Therapeutic Guidelines (electronic)
- ☐ UpToDate
- ☐ Local hospital clinical guidelines
- ☐ Best Practice
- ☐ Pharmaceutical Benefits Scheme (PBS)
- ☐ eMedicine.com (Medscape)
- ☐ Royal Children's Hospital Guidelines
- ☐ Wheeless' Textbook of Orthopaedics (online)
- ☐ TOXINZ
- ☐ Wikipedia
- ☐ Random Google search
- ☐ PubMed or Google Scholar
- ☐ Subscription Publishing Databases
- ☐ Australian Injectable Drugs Handbook
- ☐ Others (please list)

8. Please rate the following resources in terms of your usage frequency.

	Used daily	Often used	Sometimes used	Rarely used	Never used
Local hospital clinical guidelines	☐	☐	☐	☐	☐
Mims Online	☐	☐	☐	☐	☐
Australian Medicines Handbook	☐	☐	☐	☐	☐
Therapeutic Guidelines (electronic)	☐	☐	☐	☐	☐
UpToDate	☐	☐	☐	☐	☐
Research Databases	☐	☐	☐	☐	☐
Wikipedia/Google search	☐	☐	☐	☐	☐

Resource Reliability

9. How reliable do you consider the recommended therapy from the following clinical resources?

Please rate on a 1 to 5 scale, where 5 is very reliable and 1 is not reliable.

	5<br>Very reliable	4	3	2	1<br>Not reliable
Google search results	☐	☐	☐	☐	☐
Therapeutic Guidelines	☐	☐	☐	☐	☐
UpToDate	☐	☐	☐	☐	☐
Online textbooks and research articles	☐	☐	☐	☐	☐
Wikipedia	☐	☐	☐	☐	☐

10. Please make any comments about access to online clinical resources and your role as a junior doctor?
